# Supplementary material for: Reconciling Mining with the Conservation of Cave Biodiversity: A Quantitative Baseline to Help Establish Conservation Priorities
Source: PLoS One. 2016 Dec 20;11(12):e0168348. doi: 10.1371/journal.pone.0168348 (PMC5173368; doi:10.1371/journal.pone.0168348)
Supplement: S1 Dataset — (ZIP) [file pone.0168348.s002.zip › Taxa/Serra Norte/SN_2013/N4E/Lista N4E-77.pdf]

CAVIDADE N4E-0077

Classe	Ordem	Fam/Outros	Gên/Outros	Espécie	S-U	U-S	Zon.
Arachnida	Acari	(Oribatida)		sp.3	X	X	E/P
Arachnida	Acari	(Parasitengonina)		sp.6		X	P
Arachnida	Acari	Eupodidae		sp.1	X	X	E/P
Arachnida	Acari	Ixodidae	*Amblyomma*	sp.1		X	P
Arachnida	Acari	Johnstonianidae		sp.68	X		P
Arachnida	Acari	Mesostigmata		jovem	X		P
Arachnida	Acari	Prostigmata		sp.49	X		P
Arachnida	Acari	Trombiculidae		sp.39		X	P
Arachnida	Acari	Trombiculidae		sp.41	X		P
Arachnida	Amblypygi	Charinidae	*Charinus*	sp.1	X	8	P
Arachnida	Amblypygi	Phrynidae	*Heterophrynus*	*longicornis*	5		E/P
Arachnida	Araneae	Caponiidae		sp.1		X	P
Arachnida	Araneae	Corinnidae	*Creugas*	sp.1	1	1	E/P
Arachnida	Araneae	Ctenidae		jovem	1		P
Arachnida	Araneae	Drymusidae		jovem	X		E
Arachnida	Araneae	Ochyroceratidae	*Speocera*	sp.1	X	X	P
Arachnida	Araneae	Pholcidae		jovem	X		P
Arachnida	Araneae	Theridiidae		jovem	X		E
Arachnida	Araneae	Theridiosomatidae	*Plato*	sp.1	X	X	E/P
Arachnida	Opiliones	Escadabiidae		sp.2	X		P
Arachnida	Opiliones	Gonyleptidae	*Spinopilar*	sp.1		X	
Arachnida	Opiliones	Stygnidae	*Protimesius*	*gracilis*		1	P
Arachnida	Palpigradi	Eukoeneniidae	*Eukoenenia*	sp.1		X	E/P
Arachnida	Pseudoscorpiones	Bochicidae		sp.1	X	X	P
Arachnida	Pseudoscorpiones	Chthoniidae	*Pseudochthonius*	sp.1	X	X	E/P
Arachnida	Schizomida	Hubbardiidae	aff. *Rowlandius*	sp.1	X	X	E/P
Chilopoda	Geophilomorpha	Ballophilidae	*Ityphilus*	sp.1		1	P
Chilopoda	Scolopendromorpha	Cryptopidae	*Cryptops*	sp.1	1	1	P
Chilopoda	Scutigeromorpha	Pselliodidae		jovem	1		P
Diplopoda	Polydesmida	Chelodesmidae		sp.3		2	P
Diplopoda	Polydesmida	Fuhrmannodesmidae		sp.1		X	P
Diplopoda	Polydesmida	Paradoxosomatidae		jovem		1	P
Diplopoda	Polydesmida	Pyrgodesmidae		sp.1		X	E/P
Entognatha	Collembola	Isotomidae		sp.1	X		P
Entognatha	Collembola	Paronellidae		sp.1		X	E/P
Entognatha	Collembola	Paronellidae		sp.4	X	X	P
Entognatha	Collembola	Sminthuroidea		sp.2		X	E/P
Entognatha	Diplura	Campodeidae		sp.1	X	X	E/P
Insecta	Blattodea			jovem	4	1	E/P
Insecta	Coleoptera	Scydmaenidae		sp.3		X	P
Insecta	Coleoptera	Staphylinidae	Pselaphinae	sp.35		X	P
Insecta	Coleoptera			jovem	X	X	E/P
Insecta	Diptera	Psychodidae	Phlebotominae	sp.	X	X	E/P
Insecta	Diptera	Sciaridae		sp.	X	X	E/P
Insecta	Diptera	Tachinidae		sp.		X	E/P
Insecta	Heteroptera	Cydnidae		sp.	X	X	E/P
Insecta	Heteroptera	Dipsocoroidea		sp.	X	X	P
Insecta	Heteroptera	Reduviidae	Triatominae	sp.	2	1	E/P
Insecta	Homoptera	Cixiidae		jovem	X	X	E/P

Insecta	Hymenoptera	Formicidae	*Pachycondyla*	sp.2	X	X	E/P
Insecta	Hymenoptera	Formicidae	*Paratrechina*	sp.2	X		P
Insecta	Hymenoptera	Formicidae	*Solenopsis*	sp.1		X	E/P
Insecta	Hymenoptera	Formicidae	*Trachymyrmex*	sp.1	X	X	P
Insecta	Isoptera	Termitidae	*Nasutitermes*	sp.	X	X	E/P
Insecta	Lepidoptera	Noctuoidea		sp.5	1		P
Insecta	Lepidoptera			jovem		X	E/P
Insecta	Orthoptera	Phalangopsidae	*Phalangopsis*	sp.	86	50	E/P
Insecta	Psocoptera	Epipsocidae	*Epipsocus*	sp.2	X		E
Insecta	Thysanura	Nicoletiidae		sp.1		X	E/P
Gastropoda	Pulmonata	Bulimulidae	*Naesiotus*	sp.	X	X	P
Gastropoda	Pulmonata	Subulinidae		sp.3	X	X	E/P
Gastropoda	Pulmonata	Subulinidae		sp.5		X	P
Gastropoda	Pulmonata	Systrophiidae	*Happia*	sp.	X	X	E/P
Malacostraca	Isopoda	Armadillidae		sp.1	X		E
Malacostraca	Isopoda	Dubioniscidae		sp.1		X	E/P
Malacostraca	Isopoda	Philosciidae		sp.1	X	X	E/P
Malacostraca	Isopoda	Scleropactidae		sp.1		X	E/P
Nematoda				sp.1		X	P
Symphyla		Scutigerellidae		sp.	X		P
Amphibia	Anura	Strabomantidae	*Pristimantis*	cf. *fenestratus*	1		E
Mammalia	Chiroptera	Phyllostomidae	*Carollia*	*perspicillata*	3	10	E/P
